# Rhabdomyolysis Associated with Influenza A Virus Infection

**DOI:** 10.7759/cureus.2786

**Published:** 2018-06-11

**Authors:** Abdisamad M Ibrahim, Sukesh Manthri, Paolo K Soriano, Vikrampal Bhatti, Chaitanya K Mamillapalli

**Affiliations:** 1 Internal Medicine, Southern Illinois University School of Medicine, Springfield, USA; 2 Fellow, Saint Louis University, St. Louis, USA; 3 Department of Endocrinology, Springfield Clinic, Springfield, USA

**Keywords:** rhabdomyolysis, influenza a

## Abstract

Influenza A associated with rhabdomyolysis has become more commonly recognized in recent years. It requires prompt recognition and treatment in order to prevent heme pigment-induced acute kidney injury. Here we report a 50-year-old female without a significant past medical history who presented with a one-week history of fevers, chills, fatigue, and generalized body aches. She was on no prior medication. Laboratory studies were significant for leukocytosis and elevated creatinine kinase up to a peak of 28,216 IU/L. Rapid influenza antigen testing was positive for influenza A virus. The patient was diagnosed with influenza A-induced rhabdomyolysis. According to our literature review, we are the first to report a case of influenza A-induced rhabdomyolysis in the 2017-2018 flu season. This case highlights the importance of considering rhabdomyolysis as a manifestation of an influenza infection.

## Introduction

Influenza virus continues to be a global problem every year. According to the Centers for Disease Control and Prevention (CDC), influenza affects between 9.2 and 60.8 million people in the United States every year. The virus kills between 12,000 and 56,000 annually and 140,000 to 710,000 people get hospitalized [1]. Influenza A commonly causes mild respiratory infections. However, in severe cases, influenza A can cause an acute respiratory distress syndrome, myocarditis, pericarditis, encephalitis, and rhabdomyolysis. There have been about a dozen cases reported in the literature on influenza A causing rhabdomyolysis, with the majority of them from the 2009 H1N1 epidemic [2]. The early recognition and treatment of rhabdomyolysis are important in preventing heme pigment-induced acute kidney injury. The following report documents an episode of acute rhabdomyolysis associated with influenza A virus infection.

## Case presentation

A 50-year-old female with no significant past medical history presented with a one-week history of fevers, chills, generalized body aches, and dark brown urine. She also complained of fatigue, vomiting, and diarrhea for two days prior to admission. She had no known history of liver, kidney, or muscle disorders. She denied any recent history of trauma, immobility (travel, surgery), seizures, new medications, or drug abuse. On presentation, she appeared in moderate distress. Her vital signs were as follows: BP 127/58 mmHg, heart rate (HR) 126/min, respiratory rate (RR) 28/min, maximum temperature (Tmax) 39.3 C, and oxygen saturation (O_2_ sat) 93% on room air (RA).

The physical examination demonstrated signs of dehydration, clear breath sounds, and mild generalized muscle tenderness on palpation.

The initial laboratory evaluation shows a hemoglobin of 11.3 g/dl. Elevated creatine kinase (CK) had a peak of 28,216 u/L. Rapid influenza antigen testing was positive for influenza A virus and negative for influenza B. Urinalysis showed dark brown urine, specific gravity (sp.gr.) 1.005, large occult blood, elevated protein - 30, red blood cells (RBC) < 1, no casts. Urine toxicology was negative for salicylates, acetaminophen, cocaine, marijuana, benzodiazepines, and alcohol. The rest of the labs were unremarkable (Table 1).

**Table 1:** Laboratory evaluation AST: aspartate aminotransferase; ALT: alanine aminotransferase; PT: prothrombin time; PTT: partial thromboplastin time

Total Creatine Kinase – 28216 (29-168 u/L)
Hemoglobin – 11.3 (12-16 g/dl)
Potassium – 3.5 (3.5-5 mmol/L)
Calcium – 8.6 (8.4-10.1 mg/dl)
Magnesium – 1.7 (1.6-2.6 mg/dl)
AST – 45 (5-35 u/L)
ALT – 27 (0-55 u/L)
PT – 14.6 (11.9 - 14.9 sec)
PTT – 46 (22.9 - 35.1 sec)
INR 1.2 (0.9 - 1.1)

The differential diagnosis of rhabdomyolysis can be divided into two categories: traumatic or nontraumatic. The traumatic causes of rhabdomyolysis can be due to motor vehicle accidents, prolonged immobilization, seizures, and compartment syndrome. Nontraumatic causes can be due to hyperthermia, infections, drugs, toxins, medications, metabolic myopathy, or electrolyte abnormalities. In the case of our patient, she didn’t have any trauma before her coming to the hospital and she tested negative for drugs, toxins, and electrolyte derangements. She was not on any medications that could cause rhabdomyolysis. The only possible explanation for her rhabdomyolysis was due to infections, and she tested positive for influenza A. Unfortunately, a muscle biopsy was not performed to rule out metabolic myopathy or polymyositis. However, the clinical presentation and the temporal relationship of her CK levels with influenza resolution strongly suggests a diagnosis of influenza A-induced rhabdomyolysis.

She was started on aggressive fluid resuscitation with normal saline in order to prevent heme pigment-induced acute kidney injury. She was also given oseltamivir 75 mg twice daily. Alkaline diuresis with the administration of sodium bicarbonate should be considered in the treatment of rhabdomyolysis. In our case, the patient's urine pH was 7.0 and alkaline diuresis was not indicated. After three days of hospital stay, her serum creatinine kinase downtrended to 6160, and the patient was discharged home in a stable condition.

## Discussion

Rhabdomyolysis is a common disorder seen in hospitalized patients. It might occur due to trauma, hyperthermia, exertion, metabolic myopathy, electrolyte disorders, drugs, and bacterial or viral infections. It is a life-threatening condition that occurs from damage to skeletal muscle, resulting in the release of toxic contents. Rhabdomyolysis due to viral infections, particularly influenza A, should be included in the differential diagnosis in patients coming to the hospital with flu-like symptoms who have prominent body aches, especially with the current flu epidemic. Rhabdomyolysis requires prompt recognition and treatment in order to prevent heme pigment-induced acute kidney injury.

A study of 18 cases in the 2009 influenza epidemic found that 62% of the patients had elevated creatinine kinase levels. An increase in creatinine kinase levels was also correlated with the severity of patients illness [3]. The mechanism of rhabdomyolysis-induced influenza A infection is multifactorial and has been hypothesized to occur: (1) Direct viral invasion of skeletal muscles; (2) a release of cytokines causing direct muscle damage; and (3) viral toxin causing myonecrosis leading to rhabdomyolysis [2]. Influenza A is more prevalent than influenza B, thus, there were more cases reported of influenza A-induced rhabdomyolysis. Our patient had a peak CK of 28,216 IU/L and thereafter showed a steep decline of CK levels within four days. In those patients without continued muscle injury, this is the natural course of the disease. CK has a serum half-life of about 1.5 days and declines at a relatively constant rate of about 40 to 50 percent of the previous days' value [4-6].

## Conclusions

As influenza continues to spread globally and affects more people, we are recognizing more clinical manifestations of the virus.  According to our literature review, we are the first to report a case of influenza A-induced rhabdomyolysis in the 2017-2018 flu season. This case highlights the importance of considering rhabdomyolysis associated with an influenza infection.
